# Haploid Genetic Screens Identify an Essential Role for PLP2 in the Downregulation of Novel Plasma Membrane Targets by Viral E3 Ubiquitin Ligases

**DOI:** 10.1371/journal.ppat.1003772

**Published:** 2013-11-21

**Authors:** Richard T. Timms, Lidia M. Duncan, Iva A. Tchasovnikarova, Robin Antrobus, Duncan L. Smith, Gordon Dougan, Michael P. Weekes, Paul J. Lehner

**Affiliations:** 1 Cambridge Institute for Medical Research, Addenbrooke's Hospital, Cambridge, United Kingdom; 2 Paterson Institute for Cancer Research, University of Manchester, Withington, Manchester, United Kingdom; 3 Wellcome Trust Sanger Institute, Wellcome Trust Genome Campus, Cambridge, United Kingdom; Oregon Health and Science University, United States of America

## Abstract

The Kaposi's sarcoma-associated herpesvirus gene products K3 and K5 are viral ubiquitin E3 ligases which downregulate MHC-I and additional cell surface immunoreceptors. To identify novel cellular genes required for K5 function we performed a forward genetic screen in near-haploid human KBM7 cells. The screen identified proteolipid protein 2 (PLP2), a MARVEL domain protein of unknown function, as essential for K5 activity. Genetic loss of PLP2 traps the viral ligase in the endoplasmic reticulum, where it is unable to ubiquitinate and degrade its substrates. Subsequent analysis of the plasma membrane proteome of K5-expressing KBM7 cells in the presence and absence of PLP2 revealed a wide range of novel K5 targets, all of which required PLP2 for their K5-mediated downregulation. This work ascribes a critical function to PLP2 for viral ligase activity and underlines the power of non-lethal haploid genetic screens in human cells to identify the genes involved in pathogen manipulation of the host immune system.

## Author Summary

Viruses manipulate the cellular machinery of the host to facilitate their replication and evade the host immune response. Kaposi's sarcoma-associated herpesvirus (KSHV), a gammaherpesvirus linked to the development of Kaposi's sarcoma, encodes two viral E3 ubiquitin ligases K3 and K5 which target multiple cell surface immunoreceptors for destruction. Here we employ a novel genetic screen in the haploid human cell line KBM7 to identify cellular proteins required for K5 function. This revealed an essential role for the poorly characterised protein proteolipid protein 2 (PLP2); K3 and K5 hijack PLP2 to facilitate their export out of the endoplasmic reticulum, which is necessary for ubiquitination and subsequent degradation of their substrates. Furthermore we identified many new cell surface receptors targeted by K5, all of which are likely to be dependent on PLP2. Therefore, PLP2 is likely to be a key host factor to allow KSHV immune evasion. Overall this work provides further insight into the function of this family of viral E3 ubiquitin ligases and paves the way for further study of the role of PLP2 in normal cellular function.

## Introduction

Manipulation of the cellular machinery of the host by viruses is essential to ensure their successful replication. This is particularly clear in the interactions between viruses and the immune system, as demonstrated by the large DNA viruses which encode multiple genes that manipulate the cell surface expression of many different immunoreceptors [1]. K3 and K5 are two genes encoded by Kaposi's sarcoma-associated herpesvirus (KSHV) which were originally identified through their ability to degrade major histocompatibility complex class I (MHC-I) molecules [2], [3]. These genes encode membrane-bound E3 ubiquitin ligases, which use their N-terminal RING-CH domain to direct the polyubiquitination and subsequent endolysosomal degradation of target immunoreceptors [4]. Although K3 seems primarily focussed on MHC-I, K5, with which it shares 40% amino acid identity, is more promiscuous and targets a variety of additional cell surface immunoreceptors for degradation. These include the NKT cell ligand CD1d [5], the MHC-I-related molecule HFE [6], the co-stimulatory molecule B7-2 [7], the adhesion molecules ICAM-1 [7], PECAM [8] and ALCAM [9], the NK cell ligands MICA, MICB and AICL [10], and the cellular restriction factor tetherin [11], [12]. How a single ligase is able to target such a structurally diverse range of molecules for degradation, whilst retaining specificity, is not well understood.

Although microscopy localises both K3 and K5 to the endoplasmic reticulum (ER) [2], substrate ubiquitination occurs in the late secretory pathway, including the plasma membrane [13], [14]. K3 and K5 must therefore traffic through the secretory pathway to the plasma membrane, where recruitment of serial E2 conjugating enzymes by the viral RING-CH domain leads to lysine-63-linked (in the case of K3) [15], or mixed lysine-11- and lysine-63-linked (in the case of K5) polyubiquitin chain formation and the ESCRT-mediated endolysosomal degradation of target immunoreceptors [14], [16]

To further elucidate cellular genes required for K5 function, we took advantage of the recent development in forward genetic screens in the near-haploid human KBM7 cell line. Forward genetic analysis, the concept of starting with a biological process and proceeding through to gene discovery, has a proven track record of elucidating gene function, particularly in yeast. This approach has been challenging to apply to cultured mammalian cells, owing to the difficulty in generating bi-allelic mutations in diploid cells. This problem has recently been circumvented with the demonstration that the near-haploid KBM7 cell line can be used to perform genetic screens in cultured human cells [17]. KBM7 cells were originally isolated from a patient with chronic myeloid leukaemia [18] and are haploid apart from disomy of chromosome 8 and the sex chromosomes [19]. Insertional mutagenesis of these cells with a gene-trap retrovirus generates a library of knockout cells [20], which can then be screened for individual mutants defective in the cellular process under investigation.

Thus far this technique has been applied principally to lethality-based screens to study the mechanism of action of cytotoxic drugs, bacterial toxins and viruses which kill KBM7 cells [17], [20]–[26]. In a proof-of-concept experiment, we recently showed that non-lethal haploid genetic screens could be successfully performed in KBM7 cells. The phenotypic enrichment of mutagenized KBM7 haploid cells displaying an altered cell surface phenotype by fluorescence-activated cell sorting (FACS) allowed us to identify known components of the MHC-I antigen presentation pathway [27]. Here we extend this approach by showing that non-lethal genetic screens in KBM7 cells can also be performed using integrated transgenes and allowed us to identify a function for the uncharacterised proteolipid protein 2 (PLP2). We show an absolute requirement for PLP2 to mediate the export of the K3 and K5 viral E3 ligases out of the endoplasmic reticulum, and consequently to allow their ubiquitinating activity. Furthermore, using our recently developed proteomic technique ‘plasma membrane profiling’ [28], we identify an additional 74 targets of K5, all of which are likely to be dependent on PLP2. This ability to selectively enrich mutant cells by cell sorting together with the use of reporter constructs enables non-lethal haploid genetic screens to be used to identify the genetic components of essentially any cellular process.

## Results

### A haploid genetic screen identifies PLP2 as a cellular gene required for the function of K5

**Figure 1:**
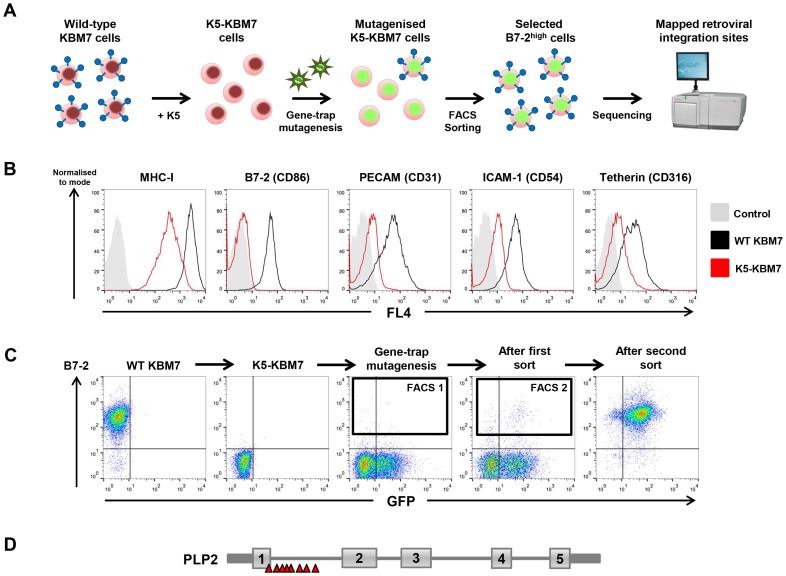
A haploid genetic screen identifies a requirement for the PLP2 gene in the K5-mediated downregulation of B7-2. (**A**) Schematic view of the genetic screen, designed such that knockout of a gene essential for K5 function in K5-KBM7 cells would rescue cell surface expression of K5 targets. (**B**) K5 is active in KBM7 cells. Wild-type (WT) KBM7 cells (black line) and K5-KBM7 (red line) were labelled for the indicated K5 target proteins and analysed by flow cytometry. (**C**) Selecting mutant B7-2^high^ cells by FACS. Near-haploid K5-KBM7 cells were mutagenised with gene-trap retrovirus and rare B7-2^high^ cells selected by two sequential rounds of FACS. Approximate sorting gates are indicated. (**D**) Sequencing of retroviral insertion sites in the selected B7-2^high^ population revealed 14 independent insertions in the PLP2 gene on the X chromosome.

To identify novel host factors required for the function of the KSHV-encoded E3 ubiquitin ligase K5, we performed a forward genetic screen in near-haploid KBM7 cells. The rationale for this approach was our prediction that inactivation of gene(s) essential for K5 function in K5-KBM7 cells would rescue the surface expression of K5 target proteins to wild-type levels (**Figure 1A**). Lentiviral expression of K5 in KBM7 cells decreased cell surface expression of known K5 targets, including MHC-I, B7-2, ICAM-1, PECAM and tetherin (**Figure 1B**). We chose to screen on the most downregulated cell surface target B7-2, which was barely detectable on the surface of K5-expressing KBM7 cells (**Figure 1B**). Mutagenesis of K5-KBM7 cells with gene-trap retrovirus (**Figure S1**) generated rare B7-2^high^ cells, which were selectively enriched from the bulk population of B7-2^low^ cells via two rounds of FACS for high surface B7-2 expression (**Figure 1C**). Following the second sort a relatively pure population of B7-2^high^ cells was established, from which genomic DNA was extracted. Retroviral integration sites were then mapped using splinkerette-PCR followed by 454 pyrosequencing [29], which revealed 14 independent retroviral insertions on the X chromosome in the gene encoding proteolipid protein 2 (PLP2, also known as protein A4) (**Figure 1D** **and Figure S2**). No other clusters of retroviral insertion sites were found elsewhere in the genome, and as such PLP2 represents the only *bona fide* hit from the genetic screen.

### PLP2 is required for the function of K5 and K3, but not the cellular MARCH proteins

**Figure 2:**
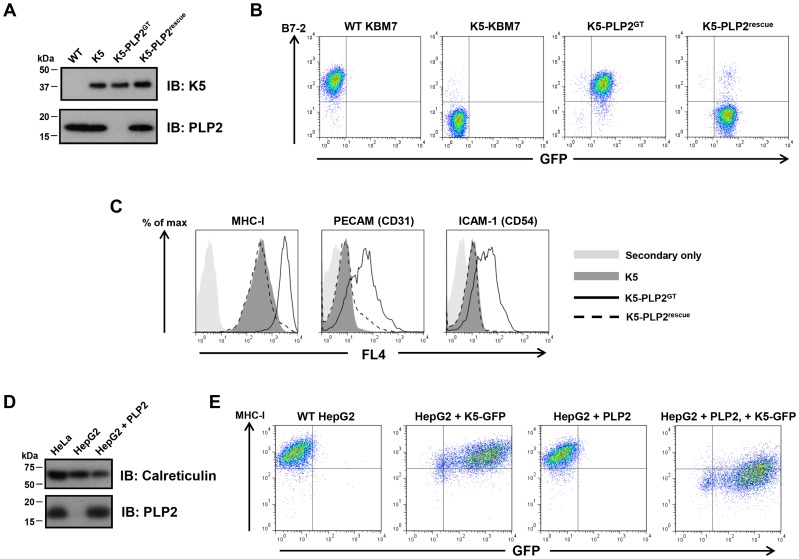
PLP2 is required for the function of K5. (**A**) PLP2 protein is not expressed in K5-PLP2^GT^ cells. K5 and PLP2 were analysed by immunoblot from cell lysates of the indicated cell lines. (**B**) PLP2 is required for the K5-mediated downregulation of B7-2 in KBM7 cells. The K5-PLP2^GT^ clone was transduced with a lentiviral vector expressing PLP2. Untransduced cells were removed by puromycin selection, and the surface expression of B7-2 in the indicated cell types was analysed by flow cytometry. Re-expression of PLP2 in K5-PLP2^GT^ cells restores the K5-mediated downregulation of B7-2. Incomplete puromycin selection means that a small population of cells remain that are not transduced with PLP2, which therefore do not recover the K5-mediated downregulation of B7-2. (**C**) K5 requires PLP2 to downregulate a range of its targets. Cell surface expression of MHC-I, PECAM and ICAM-1 were analysed in the indicated cell types by flow cytometry. (**D**) HepG2 cells do not express detectable PLP2 protein. Immunoblot analysis of PLP2 from cell lysates of the indicated cell lines. (**E**) K5 cannot function in HepG2 cells owing to the absence of PLP2. Lentiviral expression of K5 alone in WT HepG2 cells does not affect cell surface MHC-I expression as measured by flow cytometry, but co-expression of K5 and PLP2 leads to downregulation of cell surface MHC-I.

A major advantage of this technique is that human somatic cell knockouts of the gene of interest are generated during the selection process. Single cell cloning of the enriched B7-2^high^ population identified a PLP2 gene-trap cell (K5-PLP2^GT^), which expressed no detectable PLP2 protein by immunoblot, but, importantly, did still express K5 protein (**Figure 2A**). To demonstrate that the inactivation of PLP2 was indeed responsible for the loss of K5 activity, we showed that exogenous expression of PLP2 in K5-PLP2^GT^ cells restored the K5-mediated downregulation of B7-2 (**Figure 2B**), proving that PLP2 is necessary for K5 function in KBM7 cells. PLP2 was also required for the downregulation of other known K5 targets, as the surface expression of MHC-I, ICAM-1 and PECAM were all at wild-type levels in K5-PLP2^GT^ cells; in each case, re-expression of PLP2 restored the K5-mediated downregulation (**Figure 2C**). Similar results were obtained using four other independent K5-PLP2^GT^ clones. To demonstrate that PLP2 was required for K5 function in a cell type other than KBM7, we found that K5 was unable to decrease cell surface MHC-I expression in hepatocellular carcinoma HepG2 cells, which do not express PLP2 [30] (**Figure 2D**). Co-expression of PLP2 restored K5 function in these cells, resulting in a decrease in cell surface MHC-I expression **(** **Figure 2E** **)**. The requirement for PLP2 for K5 function is therefore independent of cell type.

**Figure 3:**
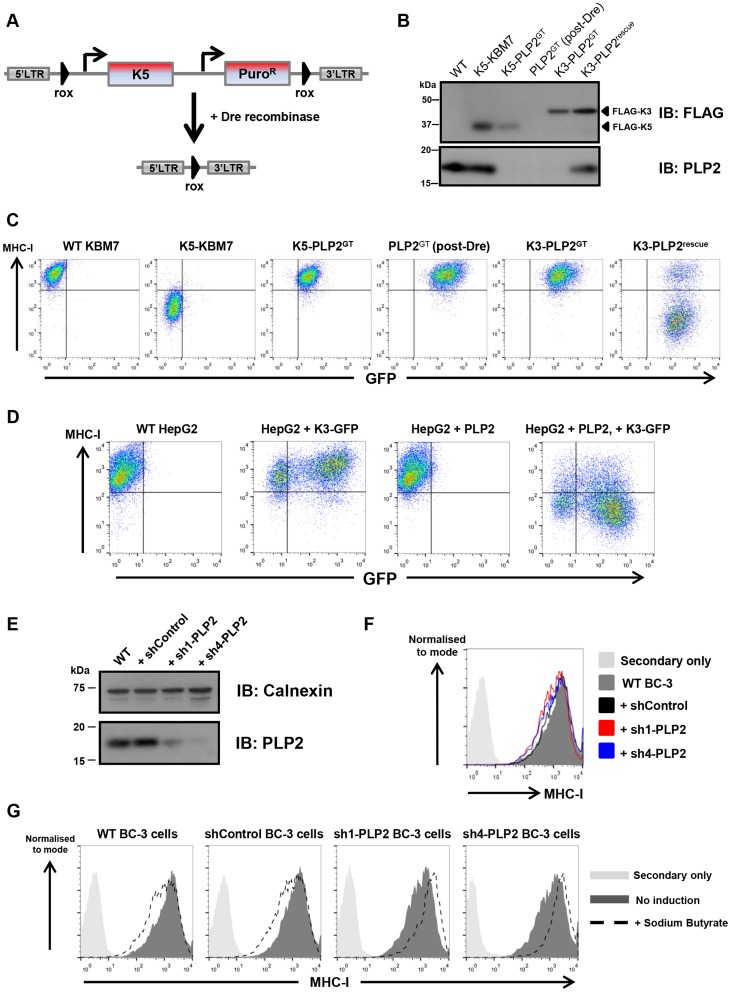
PLP2 is required for the function of K3. (**A**) Schematic representation of the dre-mediated excision of K5 to generate a ‘clean’ PLP2 knockout cell. (**B**) Excision of K5 from K5-PLP2^GT^ cells. Immunoblot analysis of FLAG-tagged K3, FLAG-tagged K5 and PLP2 from the indicated cell lines. The resulting PLP2^GT^ cells no longer express K5. (**C**) K3 requires PLP2 to downregulate MHC-I. Cell surface expression of MHC-I was analysed by flow cytometry in the indicated cells. Due to incomplete transduction with PLP2, there is a population of K3-PLP2^rescue^ cells that fail to downregulate MHC-I (right plot). (**D**) K3 function in HepG2 is rescued following expression of exogenous PLP2. Cell surface expression of MHC-I was analysed in the indicated cells. As K3 and GFP are expressed from different promoters in the expression construct, it is likely that GFP levels do not accurately reflect K3 levels in the cell, and hence even some of the GFP-negative cells have sufficient K3 to downregulate MHC-I. (**E**) Knockdown of PLP2 in BC-3 cells. BC-3 cells were transduced with lentiviral vectors encoding either a non-targeting shRNA (shControl), or shRNAs against PLP2 (sh1-PLP2 and sh4-PLP2), and transduced cells selected using puromycin. PLP2 levels in the resulting lines were analysed by immunoblot. (**F**) Knockdown of PLP2 does not affect cell surface MHC-I levels in BC-3 cells. Cell surface levels of MHC-I in the indicated cell lines were analysed by flow cytometry. (**G**) Knockdown of PLP2 prevents the downregulation of MHC-I upon reactivation of KSHV in BC-3 cells. The indicated cell lines were treated with sodium butyrate, and 24 hours later cell surface MHC-I levels were analysed by flow cytometry.

Given the essential role of PLP2 in K5 function, we wanted to determine whether it was also required for K3, and the cellular orthologues of K3 and K5, the membrane-associated RING-CH (MARCH) family of proteins [31]. This is best examined in a PLP2 knockout cell in the absence of K5, and therefore required excision of the K5 transgene from the genome of K5-PLP2^GT^ cells. To generate this cell line, we repeated the phenotypic screen, but used a K5 expression construct flanked by rox sites (**Figure 3A**). Analogous to the cre/loxP system, dre recombinase catalyses the excision of DNA flanked with rox sites [32] (**Figure S3**). The repeat screen again generated PLP2 gene-trap cells. Following dre treatment we established a clonal line (PLP2^GT^) from which K5 had been excised, as shown by the lack of K5 protein expression by immunoblot (**Figure 3B**) and the lack of any downregulation of the K5 target MHC-I upon re-expression of PLP2 (**Figure 3C**).

K3 was unable to decrease MHC-I levels when expressed in PLP2^GT^ cells, but its activity was restored upon re-expression of PLP2 (**Figure 3C**). A similar effect was also seen in HepG2 cells (**Figure 3D**). However, MARCH1, MARCH8 and MARCH9 all efficiently downregulated their cell surface targets in PLP2^GT^ cells (**Figure S4**), suggesting that PLP2 is not an essential factor for the MARCH proteins.

### PLP2 is required for MHC-I downregulation in KSHV-infected cells

K3 and K5 are responsible for the downregulation of MHC-I from the cell surface during KSHV infection [33]. Given that PLP2 is essential for the function of both K3 and K5 when the viral ligases are expressed individually in cultured cells, we would predict that PLP2 expression would also be critical for MHC-I downregulation by K3 and K5 in KSHV-infected cells. To test this hypothesis we used short-hairpin RNA (shRNA) lentiviral vectors to knockdown PLP2 expression in BC-3 cells, which are latently infected with KSHV. We designed four independent shRNA vectors against PLP2 and tested their efficacy to knockdown PLP2 expression by immunoblot (**Figure S5A**), and by their ability to inhibit the K5-mediated downregulation of MHC-I in the monocytic THP-1 cell line (**Figure S5B**). The two leading hairpins (sh1-PLP2 and sh4-PLP2), together with a non-targeting hairpin (shControl) were used to transduce BC-3 cells, and knockdown of PLP2 was confirmed by immunoblot (**Figure 3E**). PLP2 knockdown by itself did not significantly affect cell surface MHC-I levels (**Figure 3F**). Lytic reactivation of KSHV in BC-3 cells was then induced using sodium butyrate, and cell surface levels of MHC-I measured by flow cytometry (**Figure 3G**). In wild-type and shControl-transduced BC-3 cells, butyrate treatment resulted in a decrease in MHC-I levels. In contrast, MHC-I was not downregulated in either shPLP2-transduced knockdown cell line; indeed MHC-I levels actually increased, probably as a result of an interferon effect that could no longer be overcome by K3 and K5. Therefore, PLP2 is essential for the K3- and K5-mediated downregulation of MHC-I in KSHV-infected cells.

### PLP2 is a MARVEL domain-containing protein required for the ubiquitination of K3 and K5 substrates

**Figure 4:**
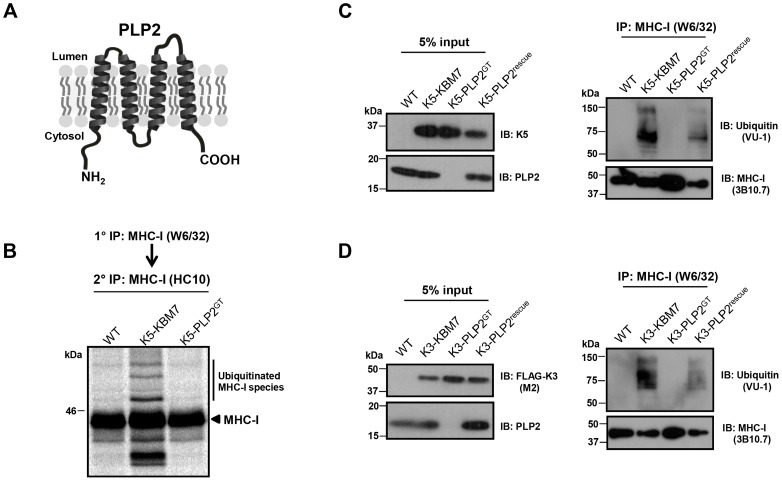
PLP2 is required for K3 and K5 to ubiquitinate their substrate MHC-I. (**A**) Schematic representation of PLP2 topology. (**B–D**) K3 and K5 are unable to ubiquitinate MHC-I in the absence of PLP2. In (**B**), the indicated cell lines were pulse-labelled for 10 minutes with [^35^S]-methionine, chased for 45 minutes, and MHC-I immunoprecipitated with the conformationally sensitive W6/32 mAb, and after dissociation and denaturation in 1% SDS, MHC-I molecules re-precipitated with the anti-MHC-I heavy chain mAb HC10, resolved by SDS-PAGE and analysed by autoradiography. In (**C**) and (**D**), MHC-I molecules were immunoprecipitated with the W6/32 mAb from the indicated cell types, and ubiquitinated MHC-I species were detected by immunoblot using the anti-ubiquitin antibody VU-1 (right panels) and the input cell lysates blotted for the indicated proteins (left panels).

PLP2 is a small 17 kDa integral membrane protein which on bioinformatic analysis contains a MARVEL (**M**AL **a**nd **r**elated proteins for **ve**sicle trafficking and membrane **l**ink) domain, characterised by an M-shaped four-membrane spanning architecture with both N- and C-terminal tails in the cytoplasm [34] (**Figure 4A**). Although the precise function of the MARVEL domain remains unclear, it is found in the myelin and lymphocyte protein (MAL), physin, gyrin and occludin protein families, some of which are implicated in vesicle trafficking and membrane apposition events [34].

To understand the requirement for PLP2 in K3 and K5 function, we determined whether PLP2 was required for the ubiquitinating activity of these viral ligases. The robust ubiquitination of MHC-I induced by both K3 and K5 in wild-type KBM7 cells, as assessed by either immunoblot or radiolabelling, was totally abrogated in the PLP2 knockout cell (**Figure 4B–C**). Therefore, PLP2 is required for the ubiquitination of K3 and K5 substrates.

### PLP2 interacts with K3 and K5 and is required for the export of a K3•MHC-I complex from the ER

**Figure 5:**
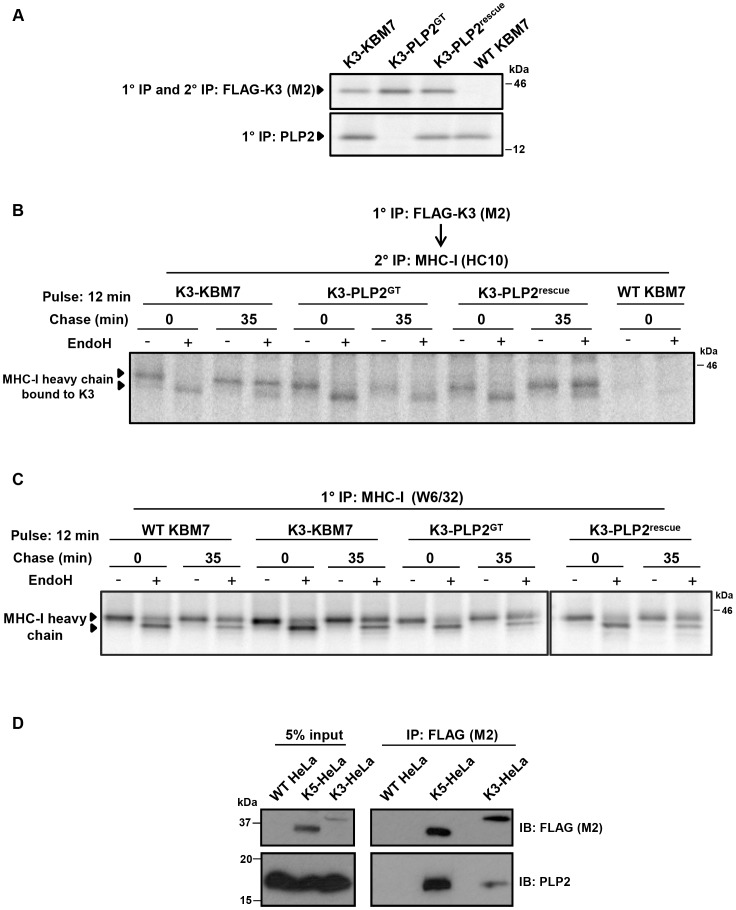
PLP2 is required for the export of a K3•MHC-I complex from the ER. (**A**) Validation of the cell lines used for the experiment. FLAG-K3 and PLP2 were immunoprecipitated from the indicated [^35^S]-methionine pulse-labelled cell types, resolved by SDS-PAGE and analysed by autoradiography. (**B**) K3-bound MHC-I remains EndoH-sensitive in the absence of PLP2. The indicated cell types were [^35^S]-methionine pulse-labelled for 12 min, chased for the indicated times and solubilised in 1% digitonin. FLAG-K3 was immunoprecipitated using the anti-FLAG M2 mAb, dissociated in 1% SDS, MHC-I molecules re-precipitated with the anti-MHC-I heavy chain mAb HC10 and then either EndoH (+) or mock digested (−) before the samples were resolved by SDS-PAGE and analysed by autoradiography. (**C**) Loss of PLP2 does not affect the normal MHC-I synthetic pathway. Free [^35^S]-methionine labelled MHC-I molecules were immunoprecipitated from the post-M2 supernatants (from B) using the W6/32 mAb, EndoH (+) or mock digested (−), and analysed by SDS-PAGE and autoradiography. (**D**) PLP2 co-immunoprecipitates with K3 and K5. FLAG-K3 and K5 were immunoprecipitated from digitonin lysates of HeLa cells using the anti-FLAG M2 mAb, and co-immunoprecipitated PLP2 detected by immunoblot.

K3 and K5 ubiquitinate their substrates in the late secretory pathway [13], [14]. The loss of ubiquitinating activity in the PLP2-knockout KBM7 cells, together with the described trafficking function of MARVEL domain-containing proteins, suggested a role for PLP2 in trafficking the viral ligases and/or their substrates to the late secretory compartment where ubiquitination can occur. However the vast majority of K3 and K5 localise to the ER [2], [35], preventing detection of any trafficking defect by microscopy. We therefore took a biochemical approach and visualised trafficking of the viral ligases through the secretory pathway using Endoglycosidase H (EndoH) sensitivity. Although K3 and K5 are not themselves glycosylated (and artificial insertion of an N-linked glycan abrogates their function, unpublished data), the K3-associated MHC-I heavy chains do contain an N-linked glycan which permits this approach. The K3-associated MHC-I which remain EndoH sensitive are localised to the ER, while K3-associated MHC-I which acquire EndoH resistance have exited the ER and trafficked beyond the medial-Golgi. By [^35^S]-methionine radiolabeling and pulse-chase analysis of K3-KBM7 cells in the presence and absence of PLP2 (**Figure 5A**) we found that K3 associated with EndoH-sensitive MHC-I at the 0 time point, irrespective of PLP2 expression (**Figure 5B**). After 35 minutes, in the presence of PLP2, almost all the K3-associated MHC-I had acquired EndoH resistance, implying that the K3•MHC-I complex had trafficked beyond the medial-Golgi (**Figure 5B** **, lane 4**). In contrast, in the PLP2-deficient cells, the K3-associated MHC-I remained exclusively EndoH-sensitive (**Figure 5B** **, lane 8**). The loss in signal of K3-associated MHC-I in the PLP2-deficient cells is probably due to the MHC-I escaping K3 and trafficking to the cell surface. These data show an essential requirement for PLP2 in the export of the K3•MHC-I complex from the ER. Importantly, these results cannot be explained by the lack of PLP2 affecting the normal maturation of MHC-I, as we observed no difference in the egress of MHC-I not bound by K3 in the presence or absence of PLP2 (**Figure 5C**). Furthermore, the requirement for PLP2 for the normal trafficking of the K3•MHC-I complex suggests that PLP2 likely interacts with K3 in the ER and facilitates its export. In support of this model, we could readily detect PLP2 bound to K3 or K5 following immunoprecipitation of FLAG-tagged K3 or K5 from HeLa cells (**Figure 5D**).

### An ER-retained PLP2 mutant cannot support K5 function

To determine the subcellular localisation of PLP2, we initially confirmed detection of endogenous PLP2 by immunofluorescence (**Figure S6A**). In HeLa cells, endogenous PLP2 concentrated primarily in a perinuclear compartment and in tubules emanating from that compartment, and expression of PLP2 tagged with mCherry at its N-terminus (mCherry-PLP2) assumed a similar distribution (**Figure S6B**). This compartment co-stained with markers of the recycling endosome, as seen with Rab11-GFP, internalised CD59 (a GPI-anchored protein that enters the cell by clathrin-independent endocytic pathways) [36], and, to some extent, the transferrin receptor (**Figure S6C**). A similar localisation of PLP2 was also observed in KBM7 cells (**Figure S6D**). Therefore PLP2 is an integral membrane protein which, following its insertion into the ER and traffic through the secretory pathway, concentrates in recycling endosomes.

**Figure 6:**
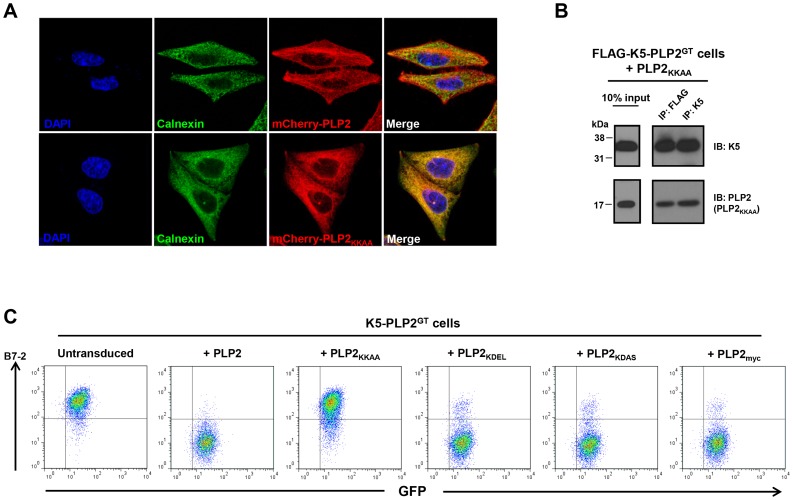
An ER-retained PLP2 mutant can still bind K5 but cannot support the K5-mediated downregulation of B7-2. (**A**) Addition of a di-lysine ER retention motif onto the C-terminal tail of PLP2 relocalises the protein to the ER. HeLa cells were transduced with lentiviral vectors expressing mCherry-PLP2 or mCherry-PLP2_KKAA_, fixed, and immunostained for calnexin to mark the ER. (**B**) K5 can still bind the PLP2_KKAA_ mutant. K5 was immunoprecipitated from K5-PLP2^GT^ cells expressing the PLP2_KKAA_ mutant, resolved by SDS-PAGE, and co-immunoprecipitating PLP2 detected by immunoblot. (**C**) The ER-retained PLP2_KKAA_ cannot support the K5-mediated downregulation of B7-2. K5-PLP2^GT^ cells were transduced with a lentiviral vector encoding the indicated PLP2 mutant proteins and analysed for cell surface B7-2 expression by flow cytometry.

If PLP2 is indeed required for the export of K3 and K5 from the ER, an ER-retained PLP2 mutant should not support K5 function. We therefore inserted a C-terminal di-lysine KKAA ER retention/retrieval motif [37] onto the C-terminus of PLP2, which resulted in its redistribution from tubular recycling endosomes to the ER in HeLa cells, as confirmed by co-localisation with the ER marker calnexin (**Figure 6A**). In K5-PLP2^GT^ cells, the PLP2_KKAA_ mutant still bound K5 (**Figure 6B**), but could not compensate for the lack of PLP2 and was unable to rescue the K5-mediated downregulation of B7-2 (**Figure 6C**). This was not merely a consequence of adding extra residues onto the C-terminal tail of PLP2, as both PLP2_KDEL_ (an ER retention signal for soluble proteins of the ER lumen) [38] and PLP2_KDAS_ rescued the K5-mediated downregulation of B7-2 in K5-PLP2^GT^ cells (**Figure 6C**). Taken together, these data support a model whereby PLP2 binds K3 and K5 in the ER and is required for their export into the late secretory pathway where the viral ligases can ubiquitinate their substrates.

### PLP2 is required for the downregulation of all K5 targets

**Figure 7:**
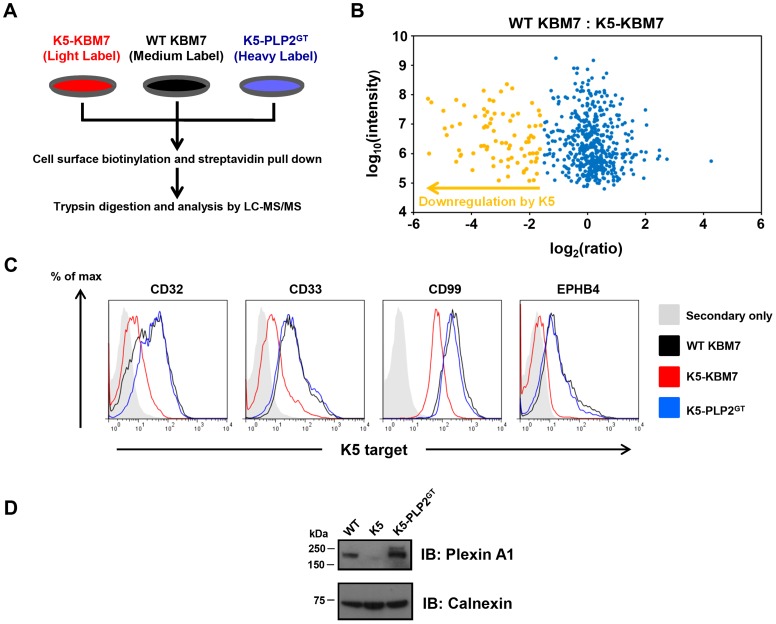
PLP2 is required for the downregulation of novel K5 targets. (**A**) Schematic view of the plasma membrane profiling (PMP) experiment. (**B**) Comparsion of the plasma membrane proteome of WT KBM7 cells versus K5-KBM7 cells. A scatterplot of proteins identified by PMP and quantified by >2 unique peptides is shown. The summed ion intensity (y-axis) is shown as log_10_. Proteins downregulated more than three-fold from the plasma membrane in the presence of K5 are highlighted in orange. A fully annotated plot is shown in Figure S5. (**C–D**) Validation of novel PLP2-dependent K5 targets. The expression of the indicated K5 targets proteins in WT KBM7 (black), K5-KBM7 (red) and K5-PLP2^GT^ (blue) were analysed by (**C**) flow cytometry and (**D**) immunoblot.

To assess what proportion of K5 targets were dependent on PLP2 expression for their K5-mediated downregulation, we performed ‘Plasma Membrane Profiling’ [28], [39], [40], a recently-developed proteomic technique which compares the relative abundance of cell surface proteins between different cell types. WT KBM7, K5-KBM7 and K5-PLP2^GT^ cells were grown in stable isotope labelling by amino acids in cell culture (SILAC) media and after selective enrichment for plasma membrane proteins, plasma membrane protein expression was quantified by mass spectrometry (**Figure 7A**). Comparison of the plasma membrane proteome of WT KBM7 versus K5-KBM7 cells identified cell surface proteins downregulated by K5, while comparison of K5-KBM7 cells versus K5-PLP2^GT^ cells identified target proteins dependent on PLP2 for their K5-mediated downregulation (**Table S1, S2, S3**).

We quantified 470 plasma membrane proteins, of which 83 were downregulated >3-fold from the plasma membrane upon expression of K5 (**Figure 7B** **, Figure S7 and Table S1**). Of these 83 target proteins, 73 (88%) were clearly dependent on PLP2 for K5-mediated downregulation (**Table S2**). These included the known K5 targets, including MHC-I, B7-2, ICAM-1, PECAM, ALCAM and the IFN-γ receptor, thereby validating the dataset (**Table S3**). However, the majority of the downregulated proteins (74/83, 89%) had not previously been identified as K5 targets. In particular, multiple members of three families of proteins were found to be downregulated by K5: receptor-type tyrosine phosphatases (PTPRA, PTPRF, PTPRK, PTPRM and PTPRS), ephrin receptors and their ligands (EPHA2, EPHB3, EPHB4; EFNB1, EFNB2 and EFNB3) and plexin receptors and their semaphorin ligands (PLXNA1, PLXNB1, PLXNC1, PLXND1; SEMA-4G). Four of the novel targets (CD32, CD33, CD99 and EPHB4) were confirmed by flow cytometry (**Figure 7C**), and one additional target, Plexin-A1, was confirmed by immunoblot (**Figure 7D**).

The mass spectrometry dataset additionally suggested a small subset of K5 targets whose degradation might be PLP2 independent (**Table S2**), and these were investigated further (**Figure S8**). Myelin protein zero-like protein 2 (MPZL2, also called epithelial V-like antigen) was most strongly downregulated by K5, in a seemingly PLP2-independent manner. Immunoblotting confirmed that MPZL2 was degraded in the presence of K5 and was not detected in K5-PLP2GT cells (**Figure S8A**). However, MPZL2 protein was readily detected in a different K5-PLP2GT clone, suggesting that its K5-mediated degradation was likely to be PLP2-dependent (**Figure S8B**). Two other seemingly PLP2-independent K5 targets, the Mast/stem cell growth factor receptor Kit and the interleukin-9 receptor were confirmed as K5 targets, but were also PLP2-dependent in a second, independent K5-PLP2GT clone (**Figure S8C**). Therefore the identification of a small number of PLP2-independent K5 targets likely represent a peculiarity of the particular K5-PLP2GT clone used, and was not generalizable. This emphasises the importance of checking the phenotypes of several clones when analysing mutants derived from KBM7 screens. We conclude that PLP2 is likely to be required for the downregulation of all K5 targets.

## Discussion

Non-lethal haploid genetic provide a novel approach to identify genes required for the downregulation of plasma membrane proteins by the viral E3 ubiquitin ligase K5. Our screens revealed an absolute requirement for the poorly-characterised protein PLP2 in the K5-mediated downregulation of B7-2 and other K5 targets. Subsequent biochemical analysis demonstrated that a lack of PLP2 prevented K3 and K5 from ubiquitinating their substrates by impairing the export of the viral ligases from the ER. By using plasma membrane profiling to compare the relative abundance of cell surface proteins in the presence and absence of K5 and PLP2 we identified 74 novel plasma membrane protein targets of K5. Almost all of these were PLP2 dependent, confirming a critical role for this protein in the downregulation of K5 target proteins.

This is the first description of the mutagenesis of near-haploid KBM7 cells coupled with phenotypic selection by FACS to identify a novel gene in a cellular process. Phenotypic enrichment by cell sorting to select rare genetic mutants provides a complementary approach to the previously described live/dead screens using KBM7 cells. We demonstrate here that haploid screens can be successfully performed using stably-integrated transgenes, and, although we used the viral gene K5, this approach is equally applicable to a fluorescent reporter. The ability to design screens based on altered expression of a genetically-encoded reporter vastly increases the number of cellular processes that can be examined using this approach. However this technique also has its limitations. Additional genes known to be required for K5 activity besides PLP2 were not identified. For example, RNAi-mediated depletion of the E2 ubiquitin-conjugating enzymes UbcH5 and Ubc13 abrogates ubiquitination by K5 [14], but these genes were not identified in our haploid screens. Given that the retroviral gene-trap vector is able to integrate in essentially all expressed genes [20], our inability to isolate mutations in the genes encoding UbcH5 and Ubc13 is likely to reflect their requirement for normal cell growth. Indeed, mutant cells need to both survive and maintain normal growth characteristics for around three weeks to emerge from the two-stage cytometry selection. Mutations that result in either cell death or a growth disadvantage will likely be lost from the final pool of selected cells.

This work describes a function for PLP2, a protein which was originally identified as enriched in colonic epithelial cells, where it was suggested to multimerise and assume characteristics of an ion channel [41]. PLP2 binds the ER-resident protein Bap31 [30] and the chemokine receptor CCR1 [42], and decreased PLP2 expression has been implicated in X-linked mental retardation [43] and melanoma metastasis [44]. However, no function is ascribed to PLP2. Our demonstration that PLP2 is required for the traffic of the K3•MHC-I complex out of the ER supports the proposed role of MARVEL domain-containing proteins in regulating membrane trafficking events [34]. The few well-characterised proteins of the MARVEL family associate with specialised cholesterol-rich membrane microdomains and regulate membrane apposition events. For example in T cells MAL is required to traffic Lck to the immunological synapse [45], while synaptophysin is involved in regulating synaptic vesicle endocytosis in neurons [46].

Why might K3 and K5 need PLP2 to escape from the ER? Such protein-assisted export has been reported for a number of other proteins. The proteolytically-inactive rhomboid iRhom2 mediates the export of TACE from the ER [47], [48], and the nucleotide-sensing toll-like receptors TLR7 and TLR9 require UNC93B1 for traffic from the ER to endolysosomes [49]. Examples can also be found in yeast, where the gene product Shr3p, notably also a small four transmembrane-spanning integral membrane protein that shares the same topology as PLP2, mediates the recruitment of amino acid permeases into transport vesicles for ER export [50].

The reported association between PLP2 and Bap31 is of special interest in this regard. Bap31 is implicated in both the degradation [30], [51] and export of proteins from the ER [52], [53], suggesting a key role as a quality control factor in protein triage in the ER. Furthermore, Bap31 binds K3 and K5 [54], suggesting that Bap31 may sequester the viral ligases in the ER such that PLP2 is required to extract them from Bap31 to facilitate export. Alternatively PLP2 may serve as a recruitment factor for the packaging of the trimeric Bap31•PLP2•K5 complex into ER exit sites. Given that we identify PLP2 in our screens rather than Bap31, the former is perhaps more likely. Of note, all three proteins are unusual in containing charged residues within their transmembrane domains, which may mediate protein associations within the hydrophobic environment of the ER membrane.

Clearly PLP2 has not evolved to facilitate the trafficking of viral ligases, and its endogenous role remains unclear. Cellular proteins may also require PLP2 to exit the ER, and we are undertaking further studies to identify such PLP2 client proteins. It was surprising that none of the MARCH proteins, the cellular orthologues of K3 and K5, were affected by the absence of PLP2 (**Figure S4**). Our previous work found PLP2 to be upregulated on the cell surface upon overexpression of MARCH9 in Sultan B cells [55], and more recently many of the MARCH proteins were also shown to bind Bap31 [54]. PLP2 may still regulate MARCH function, but another protein substitutes for PLP2 to allow MARCH-mediated downregulation of cell surface targets. Alternatively the viral ligases may exploit PLP2 to facilitate their trafficking independently of the normal endogenous role of PLP2. While our experience would suggest that viral gene products are more likely to appropriate endogenous function of cellular genes, rather than invent new ones, the steady-state localisation of PLP2 to recycling endosomes (**Figure S6**) suggests additional cellular functions for PLP2 in the endosomal system. Indeed, a recent proteomic experiment identified PLP2 as a component of clathrin-coated vesicles [56].

Our proteomic dataset underscores the remarkable ability of K5 to specifically target a wide variety of structurally diverse plasma membrane proteins for degradation. In this regard, KSHV has adopted an alternative strategy to other herpesviruses. Human cytomegalovirus, for example, encodes multiple genes which each downregulate a few cell surface ligands [57], while in the case of KSHV a single gene appears to perform this function. The selective advantage to the virus of downregulating ligands for cytotoxic T cells (MHC-I), costimulatory molecules (B7-2) or NK cell ligands (MICA and MICB) is readily explained. However, this is less clear for some of the novel K5 targets such as the ephrin signalling pathway, implicated in normal thymocyte maturation and T cell modulation [58], or semaphorin/plexin signalling, involved in immune cell migration [59]. We suggest additional, as yet to be identified, roles for these cell surface proteins and it will be interesting to see which of these new substrates are also targeted by other viral proteins.

We envisage a number of potential mechanisms by which K5 targets so many different receptors: K5 could interact directly with each of its targets, K5 could bind a common adaptor protein which provides a platform from which it can ubiquitinate its targets, or K5 could be recruited to a specialised membrane microdomain where it ubiquitinates its targets. The general requirement for PLP2 suggests that K5 uses at least a broadly similar mechanism to downregulate its targets. Given the propensity for the partitioning of MARVEL domain proteins into specialised membrane microdomains, we speculate that the role of PLP2 is not simply to export K5 from the ER, but also to traffic it to specific site(s) for target ubiquitination. Identifying common features shared between the large number of K5 targets may shed further light on this important issue.

In summary, we have found an essential role for PLP2 in facilitating the ubiquitination and degradation of cell surface immunoreceptors by the viral E3 ubiquitin ligases K3 and K5. In addition we identified 74 novel plasma membrane targets of K5, all of which are likely to be PLP2-dependent; PLP2 is therefore a critical host factor for KSHV immune evasion. Further work will be required to elucidate the normal cellular function of PLP2 and to investigate the potential for interfering with PLP2 function to modulate the pathogenesis of KSHV infection.

## Materials and Methods

### Cell culture

KBM7 cells, THP-1 cells and BC-3 cells were maintained in IMDM supplemented with 10% fetal calf serum and penicillin/streptomycin. HeLa cells and HepG2 cells were grown in RPMI 1640 plus 10% fetal calf serum and penicillin/streptomycin. For reactivation of latent KSHV, BC-3 cells were treated with 2 mM sodium butyrate for 24 hours. For SILAC analysis, KBM7 cells were grown in SILAC RPMI 1640 (Thermo Pierce), 10% dialysed FBS (JRH Biosciences), and penicillin/streptomycin. SILAC media was supplemented with either light (Arg 0, Lys 0, Sigma), medium (Arg 6, Lys 4, Cambridge Isotope Laboratories) or heavy (Arg 10, Lys 8, Cambridge Isotope Laboratories) amino acids at 50 mg/l and L-proline at 280 mg/l. Incorporation of heavy label was >98% for both arginine and lysine-containing peptides.

### Constructs and antibodies

The PLP2 expression vector pcDNA3.1-PLP2myc/his was a kind gift from Prof. Jiyoung Kim (Kyung Hee University, Korea) [42], the lentiviral expression vector pHRSIN-UCOE-EGFP a kind gift from Prof. Adrian Thrasher (University College London, UK) [45], the dre recombinase expression vector pCAGGS-Dre a kind gift from Prof. Francis Stewart (TU Dreseden, Germany) (Anastassiadis et al., 2009), and the lentirviral shRNA expression vector pHR-SIREN a kind gift from Prof. Greg Towers (University College London, UK). Primary antibodies used were as follows: mAb W6/32 (recognises conformational MHC-I), mAb HC10 (anti-MHC-I heavy chain), mAb 3B10.7 (anti-MHC-I heavy chain), mouse α-B7-2 (BU63), mouse α-ICAM1-APC (BD), mouse α-PECAM-APC (BD), mouse α-tetherin (a kind gift from G. Towers, University College London), mouse α-FLAG M2 (Sigma), rabbit α-calreticulin (Thermo), mouse α-calnexin (mAb AF8, a kind gift from M. Brenner, Harvard Medical School, USA), mouse α-SLAM (Biolegend), mouse α-ubiquitin (VU-1, Lifesensors), mouse α-CD71 (Santa Cruz), mouse α-CD59 (Santa Cruz), mouse α-Rab11 (Abcam), mouse α-CD32 (BD), mouse α-CD33 (Biolegend), mouse α-CD99 (Abcam), rabbit α-EPHB4 (R&D Systems) and rabbit α-MPZL2 (Proteintech). The mouse monoclonal antibody against K5 was a gift from Prof. Klaus Früh (Oregon Health and Science University, USA) and the rabbit polyclonal antibody against PLP2 was a kind gift from Prof. Gordon Shore (McGill University, Canada) [30]. Fluorophore-conjugated secondary antibodies were obtained from Molecular Probes, and HRP-conjugated secondary antibodies were obtained from Jackson ImmunoResearch.

### Lentiviral vectors

K5 was expressed from a modified version of the lentiviral expression vector pHRSIN-UCOE-EGFP [45]. A rox site was inserted into the EcoRI site upstream of the UCOE element, the EGFP replaced with K5 with an N-terminal FLAG tag, and a puromycin resistance cassette and additional rox site inserted downstream. Otherwise the lentiviral expression vectors pHRSIN-P_SFFV_-GFP-P_PGK_-Puro and pHRSIN-P_SFFV_-GFP-P_PGK_-Hygro were used, with the gene of interest cloned in place of GFP as a BamHI-NotI fragment. Lentivirus was produced by transfecting 293ET cells with the lentiviral vector plus the packaging plasmids pCMVΔR8.91 and pMD.G using TransIT-293 (Mirus) according to the manufacutrer's instructions. The viral supernatant was collected 48 h and 72 h later and target cells transduced by spin infection at 1800 rpm for 45 min.

For shRNA-mediated knockdown of PLP2 expression, hairpin oligonucleotides were annealed, cloned into the pHR-SIREN lentiviral vector cut with BamHI and EcoRI, and sequence verified. Lentivirus was then made as above in 293ET cells and used to transduce BC-3 cells. Hairpins were designed using Clontech's RNAi target sequence selector; the forward oligonucleotide sequences used were:

*sh1-PLP2:* 5′-GAT CCG TGG TGA TCC TGA TCT GCT TTT CAA GAG AAA GCA GAT CAG GAT CAC CAT TTT TTG -3′

*sh2-PLP2:* 5′-GAT CCG CGG TGA TTG AGA TGA TCC TTT CAA GAG AAG GAT CAT CTC AAT CAC CGT TTT TTG-3′

*sh3-PLP2:* 5′-GAT CCG TGC ACA CCA AGA TAC CAT TTT CAA GAG AAA TGG TAT CTT GGT GTG CAT TTT TTG-3′

*sh4-PLP2:* 5′-GAT CCG CGG CAA TCC TCT ACC TGA TTT CAA GAG AAT CAG GTA GAG GAT TGC CGT TTT TTG-3′

*shControl:* 5′-GAT CCG TTA TAG GCT CGC AAA AGG TTC AAG AGA CCT TTT GCG AGC CTA TAA CTT TTT TG-3′

### Haploid genetic screen

The haploid genetic screen was carried out as described [27]. Briefly, 5×10^7^ near-haploid K5-KBM7 cells were mutagenised with gene-trap retrovirus, grown for 7 days, and then sorted by FACS for high B7-2 expression. A further sort to purify the B7-2^high^ population was carried out a further 7 days later, before genomic DNA was extracted and the retroviral integration sites mapped by splinkerette-PCR and 454 pyrosequencing [27], [29].

### Flow cytometry

Cells were washed with PBS and incubated with primary antibody for 30 min at 4°C, washed once with PBS, and then incubated with fluorophore-conjugated secondary antibody for 30 min at 4°C. Following fixation with 4% PFA, samples were analysed on a FACSCalibur (BD). FACS was carried on an Influx (BD) cell sorter.

### Immunofluorescence

HeLa cells were grown overnight on glass coverslips; KBM7 cells were allowed to adhere to coverslips in serum-free media. Cells were fixed with 4% PFA, permeabilised with 0.5% Triton X-100 and then blocked for 1 h with 4% BSA dissolved in PBS+0.1% Tween-20 (PBS-T). Primary antibody was then applied for 1 h, the coverslips washed in PBS-T, and the fluorophore-conjugated secondary antibody applied for 45 min. Coverslips were mounted in Prolong anti-fade reagent (Molecular Probes) and imaged using a Nikon LSM510 laser scanning confocal microscope (Zeiss). Images were processed using Adobe Photoshop (Adobe, CA).

### Co-immunoprecipitation and immunoblotting

Cells were lysed in 1% Triton X-100 or 1% Digitonin in TBS plus 10 mM iodoacetamide (IAA) and 0.5 mM phenylmethylsulfonyl fluoride (PMSF) for 30 minutes on ice. The postnuclear supernatants were heated to 70°C in SDS sample buffer for 10 minutes, separated by SDS-PAGE, and transferred to a PVDF membrane (Millipore). Membranes were blocked in 5% milk in PBS+0.2% Tween-20, probed with the indicated antibodies, and reactive bands visualised using West Pico (Thermo Fisher Scientific). To visualise ubiquitinated MHC-I using the VU-1 antibody, membranes were first cross-linked with 0.5% glutaraldehyde and processed according to the manufacturer's instructions. For immunoprecipitation, lysates were pre-cleared with protein A and IgG-Sepharose and incubated with primary antibody and protein A-Sepharose for 2 h at 4°C. Following three washes in lysis buffer, samples were eluted in SDS sample buffer and processed as above.

### Metabolic labelling and pulse-chase

Cells were starved for 1 h in cysteine- and methionine- free media, labelled for the indicated time with [^35^S]-cysteine and [^35^S]-methionine (Amersham) and chased in media containing excess cold cysteine and methionine. Cells were then lysed in 1% digitonin plus IAA and PMSF and processed for immunoprecipitation as above. For re-precipitation, the washed primary immunoprecipitates were dissociated in 1% SDS for 1 h at 37°C, diluted 20-fold in 0.5% Triton X-100, and then re-precipitated overnight with the appropriate antibody and protein A-Sepharose beads. Samples were then washed in lysis buffer, separated by SDS-PAGE, dried and analysed by autoradiography. For EndoH treatment, the samples were split into two, and EndoH (NEB) added to one of the samples following the manufacturer's instructions. Following incubation for 1 h at 37°C, samples were analysed by SDS-PAGE and autoradiography.

### Plasma Membrane Profiling

PMP was performed as described previously [28], [40]. Briefly, 2.5×10^8^ of each SILAC-labelled cell type were pooled in a 1∶1∶1 ratio. Surface sialic acid residues were oxidized with sodium meta-periodate (Thermo) then biotinylated with aminooxy-biotin (Biotium). The reaction was quenched, and the biotinylated cells incubated in a 1% Triton X-100 lysis buffer. Biotinylated glycoproteins were enriched with high affinity streptavidin agarose beads (Pierce) and washed extensively. Captured protein was denatured with DTT, alkylated with iodoacetamide (IAA, Sigma) and digested with trypsin (Promega) on-bead overnight. Tryptic peptides were collected and fractionated (described below). Glycopeptides were eluted using PNGase (New England Biolabs).

A total of 30 µg of tryptic peptide was subjected to high pH reversed phase HPLC (HpRP-HPLC) fractionation into 124 fractions [40]. Fractions were combined into 62 samples prior to analysis with the PNGase fraction using a NanoAcquity uPLC (Waters) coupled to an LTQ-OrbiTrap XL (Thermo). Mass spectrometric analysis, database searching and data processing were performed as described previously [40]. Briefly, fractionated tryptic peptides were separated using a gradient of 3 to 25% MeCN over 20 min and to 45% MeCN by 30 min. Unfractionated PNGase peptides were separated with a gradient of 3 to 25% MeCN over 60 min and to 45% MeCN by 80 min. PNGase samples were acquired in triplicate. MS data was acquired between 300 and 2000 m/z at 60,000 fwhm. CID spectra were acquired in the LTQ with MSMS switching operating in a top 6 DDA fashion. Raw MS files were processed using MaxQuant version 1.3.0.5 [60] H. sapiens Uniprot database (downloaded 29/05/13). Significance A values were calculated and Gene Ontology Cellular Compartment (GOCC) terms added using Perseus version 1.2.0.16 (downloaded from http://maxquant.org). We assessed the number of PM proteins identified as described previously [40].

## Supporting Information

Figure S1 **Schematic view of gene-trap mutagenesis.** The gene-trap retroviral vector [17], [27] is designed such that, in theory, should it insert into an expressed gene in-frame in the desired orientation, the strong adenoviral splice acceptor site (SA) will accept splicing from the upstream exon, resulting in a GFP-fusion transcript which is terminated at the downstream polyadenylation signal (pA). The loss of expression of the downstream exons creates a knockout allele.
(TIF)

Figure S2 **454 pyrosequencing of retroviral integration sites in the selected B7-2^high^ population reveals multiple independent insertions in the PLP2 gene.** (**A**) List of 454 reads mapping to PLP2. (**B**) Graphical representation produced using Integrative Genomics Viewer (IGV).
(TIF)

Figure S3 **Dre-mediated excision of rox-flanked K5 from the genome of KBM7 cells.** (**A**) Schematic representation of the dre-mediated excision of K5. FLAG-K5 was inserted into the genome of KBM7 using a lentiviral construct bearing flanking rox sites and a puromycin resistance cassette. Dre recombinase was expressed in these cells using a lentiviral vector that also expressed Emerald-GFP from the ubiquitin promoter. Therefore, dre expression in GFP+ cells should result in recombination across the rox sites and excision of K5. (**B**) Successful excision of K5. Dre expression in K5-KBM7 cells results in the restoration of cell surface B7-2 expression in the GFP+ cells, owing to the excision of K5.
(TIF)

Figure S4 **Downregulation of cell surface receptors by MARCH proteins is unaffected by loss of PLP2.** Wild-type KBM7 cells or PLP2^GT^ cells were transduced with lentiviral vectors encoding the indicated MARCH proteins along with Emerald-GFP, and the cell surface levels of the MARCH target proteins B7-2 (MARCH1 and MARCH8) and SLAM (MARCH9) were assessed by flow cytometry.
(TIF)

Figure S5 **Validation of shRNA lentiviral vectors to knockdown PLP2 expression.** (**A**) The effect of the shPLP2 vectors on PLP2 expression as assessed by immunoblot. THP-1 cells were transduced with four independent shPLP2 lentiviral vectors, untransduced cells removed by puromycin selection, and PLP2 expression examined by immunoblot. (**B**) The effect of the shPLP2 vectors on PLP2 expression as assessed by the inhibition of the K5-mediated downregulation of MHC-I. The shPLP2 THP-1 cells from (A) were transduced with a lentiviral vector expressing K5, and the K5-mediated downregulation of MHC-I assessed by flow cytometry.
(TIF)

Figure S6 **Subcellular localisation of PLP2.** (**A**) Proving specificity of the PLP2 antibody for immunofluorescence. (**B**) Localisation of endogenous PLP2 and mCherry-PLP2 in HeLa cells. WT HeLa cells were either immunostained with the PLP2 antibody or transduced with a lentiviral vector encoding an mCherry-tagged PLP2. (**C**) PLP2 co-localises with recycling endosome markers in HeLa cells. HeLa cells were either transfected with a plasmid expressing Rab11-GFP or stained using mouse antibodies against the transferrin receptor (TfR) or CD59 (30 min antibody uptake) together with anti-mouse secondary antibodies conjugated to Alexa Fluor-488. PLP2 was stained using a rabbit antibody together with an anti-rabbit secondary antibody conjugated to Alexa Fluor-568. (**D**) PLP2 co-localises with recycling endosome markers in KBM7 cells. KBM7 cells were stained in a similar way for the indicated markers (CNX, calnexin).
(TIF)

Figure S7 **Plasma membrane profiling identifies many new K5 targets.** A fully annotated version of Figure 7B is shown; proteins downregulated >3-fold from the plasma membrane in the presence of K5 are highlighted in orange.
(TIF)

Figure S8 **Examining potential PLP2-independent K5 targets.** (**A**) Validation of the mass spectrometry data by immunoblot, confirming MPZL2 as a PLP2-independent K5 target in the K5-PLP2^GT^ clone used. (**B**) MPZL2 cannot be degraded in the absence of PLP2 in an independent K5-PLP2^GT^ clone. (**C**) Two additional putative PLP2-independent K5 targets identified, Kit and IL9R, were found to be in fact PLP2-dependent when examined by flow cytometry in an independent K5-PLP2^GT^ clone.
(TIF)

Table S1 **K5 target proteins.** List of all proteins quantified that were downregulated >3 fold in K5-KBM7 cells compared with WT KBM7 cells. Proteins quantified by a single peptide were excluded. For a given protein, coloured squares are shown to indicate a Gene Ontology annotation of: M (membrane), PM (plasma membrane), CS (cell surface), XC (extracellular), N (nucleus) and ShG (short GO term). ‘Short GO’ refers to a subset of proteins annotated by GO as integral to the membrane, but with no subcellular assignment and a short 4- or 5-part GO cellular compartment term [39]. Of all 83 proteins identified by >1 peptide, 79 (95%) were annotated PM or CS or XC or ShG. Significance A was used to estimate p-values.
(XLSX)

Table S2 **Assessing the PLP2 dependency of K5 targets.** List of proteins quantified that were downregulated >3 fold in K5-KBM7 cells compared with WT KBM7, and that were also quantified in K5-PLP2^GT^ cells. Proteins quantified by a single peptide were excluded. K5 targets were subjected to further examination if the ratio of H∶L/M∶L was <30% (‘Potentially independent’), suggesting that the target might be PLP2-independent.
(XLSX)

Table S3 **Summary of data on known K5 targets.**
(XLSX)
