# Supplementary figures and images for: Haploid Genetic Screens Identify an Essential Role for PLP2 in the Downregulation of Novel Plasma Membrane Targets by Viral E3 Ubiquitin Ligases
Source: PLoS Pathog. 2013 Nov 21;9(11):e1003772. doi: 10.1371/journal.ppat.1003772 (PMC3836740; doi:10.1371/journal.ppat.1003772)

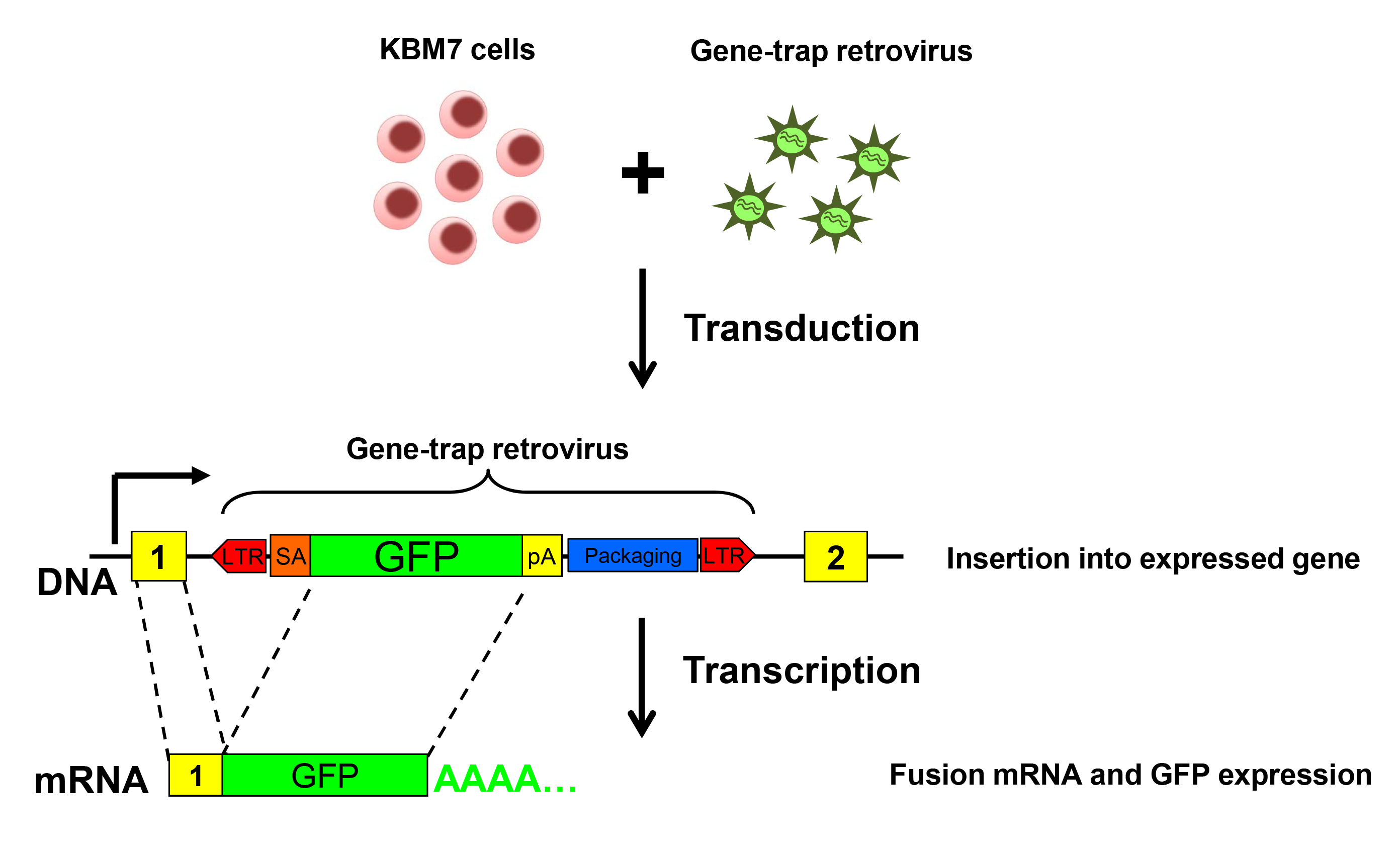

Supplement: Figure S1 — Schematic view of gene-trap mutagenesis. The gene-trap retroviral vector [17], [27] is designed such that, in theory, should it insert into an expressed gene in-frame in the desired orientation, the strong adenoviral splice acceptor site (SA) will accept splicing from the upstream exon, resulting in a GFP-fusion transcript which is terminated at the downstream polyadenylation signal (pA). The loss of expression of the downstream exons creates a knockout allele. (TIF) [file ppat.1003772.s001.tif]

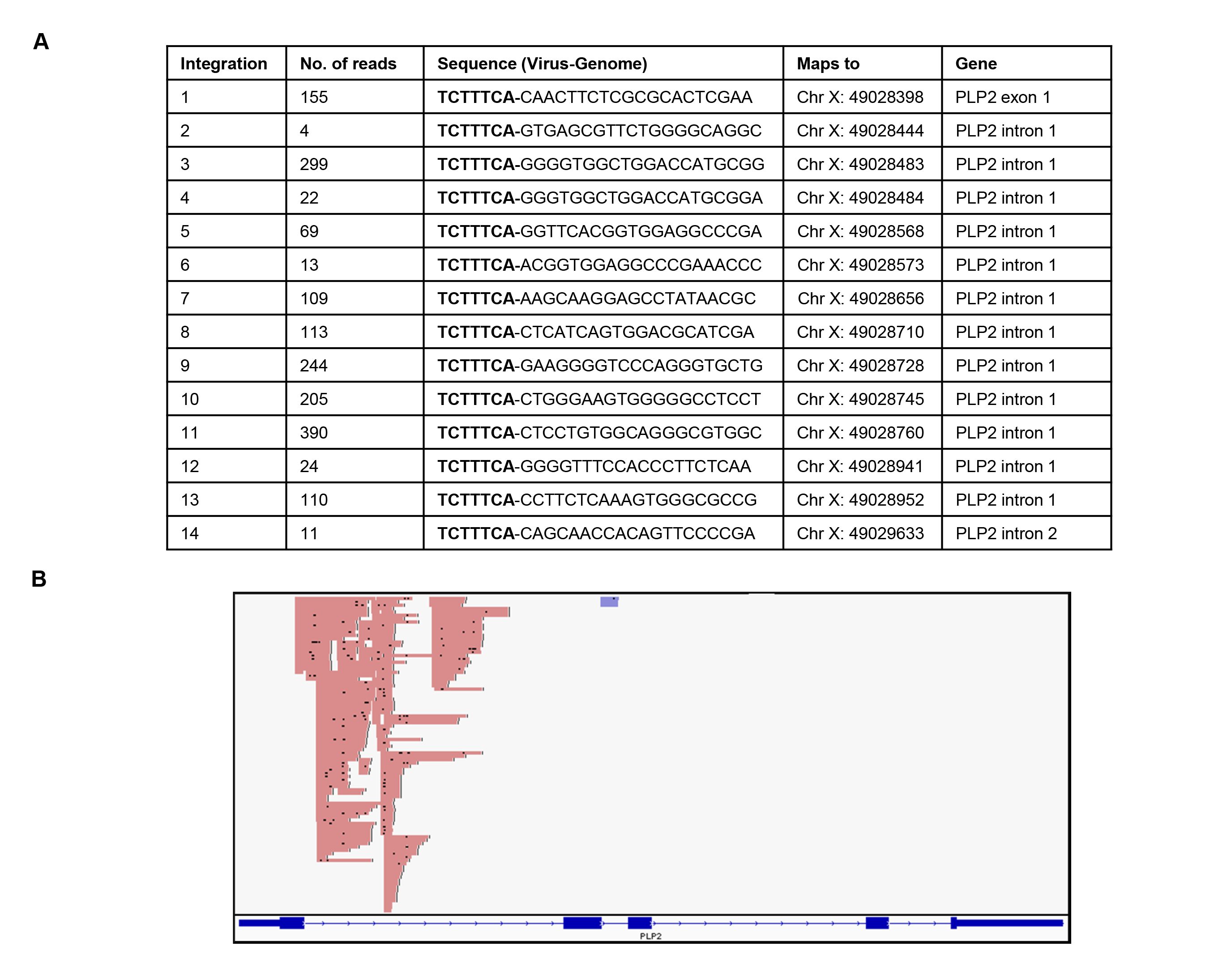

Supplement: Figure S2 — 454 pyrosequencing of retroviral integration sites in the selected B7-2high population reveals multiple independent insertions in the PLP2 gene. (A) List of 454 reads mapping to PLP2. (B) Graphical representation produced using Integrative Genomics Viewer (IGV). (TIF) [file ppat.1003772.s002.tif]

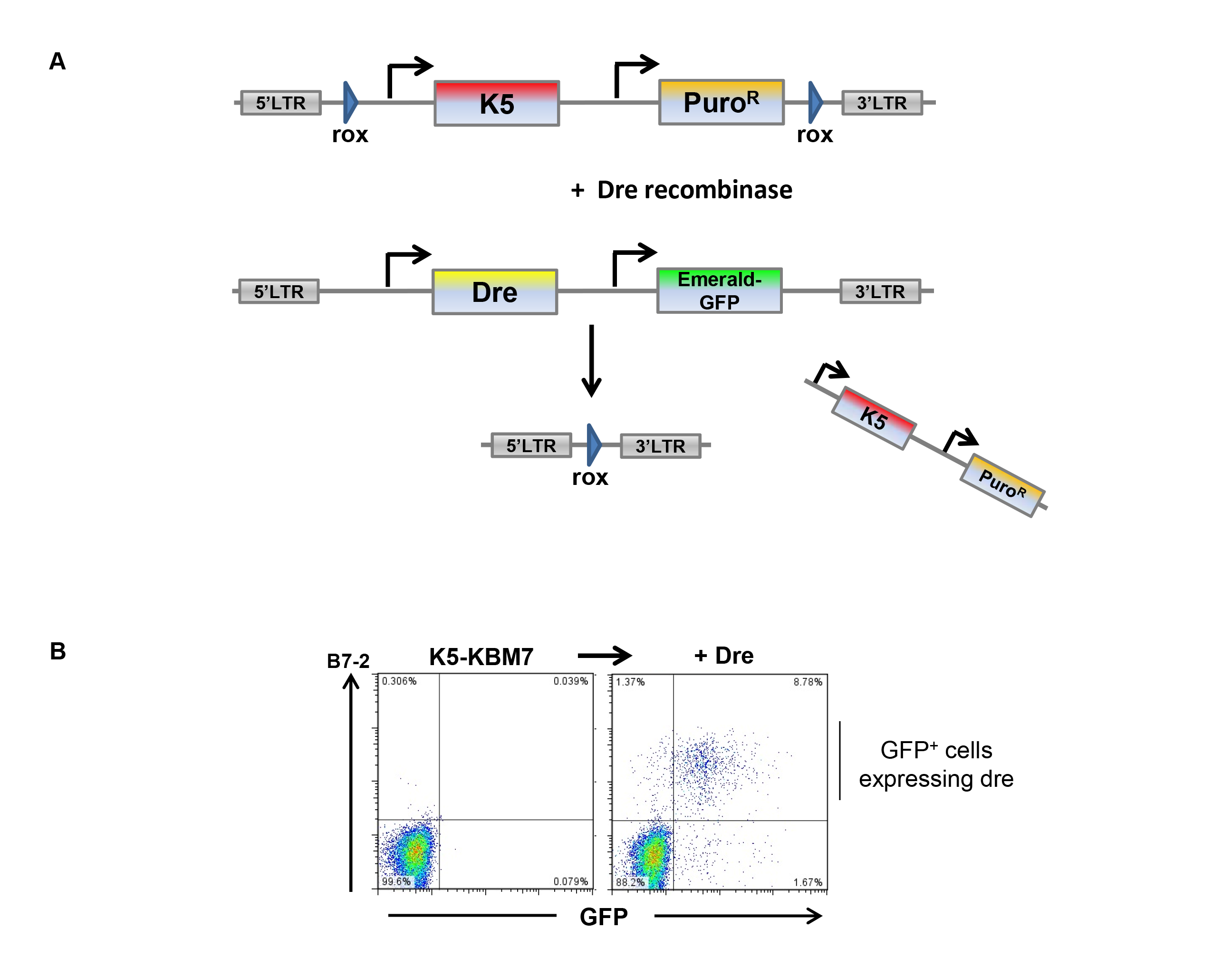

Supplement: Figure S3 — Dre-mediated excision of rox-flanked K5 from the genome of KBM7 cells. (A) Schematic representation of the dre-mediated excision of K5. FLAG-K5 was inserted into the genome of KBM7 using a lentiviral construct bearing flanking rox sites and a puromycin resistance cassette. Dre recombinase was expressed in these cells using a lentiviral vector that also expressed Emerald-GFP from the ubiquitin promoter. Therefore, dre expression in GFP+ cells should result in recombination across the rox sites and excision of K5. (B) Successful excision of K5. Dre expression in K5-KBM7 cells results in the restoration of cell surface B7-2 expression in the GFP+ cells, owing to the excision of K5. (TIF) [file ppat.1003772.s003.tif]

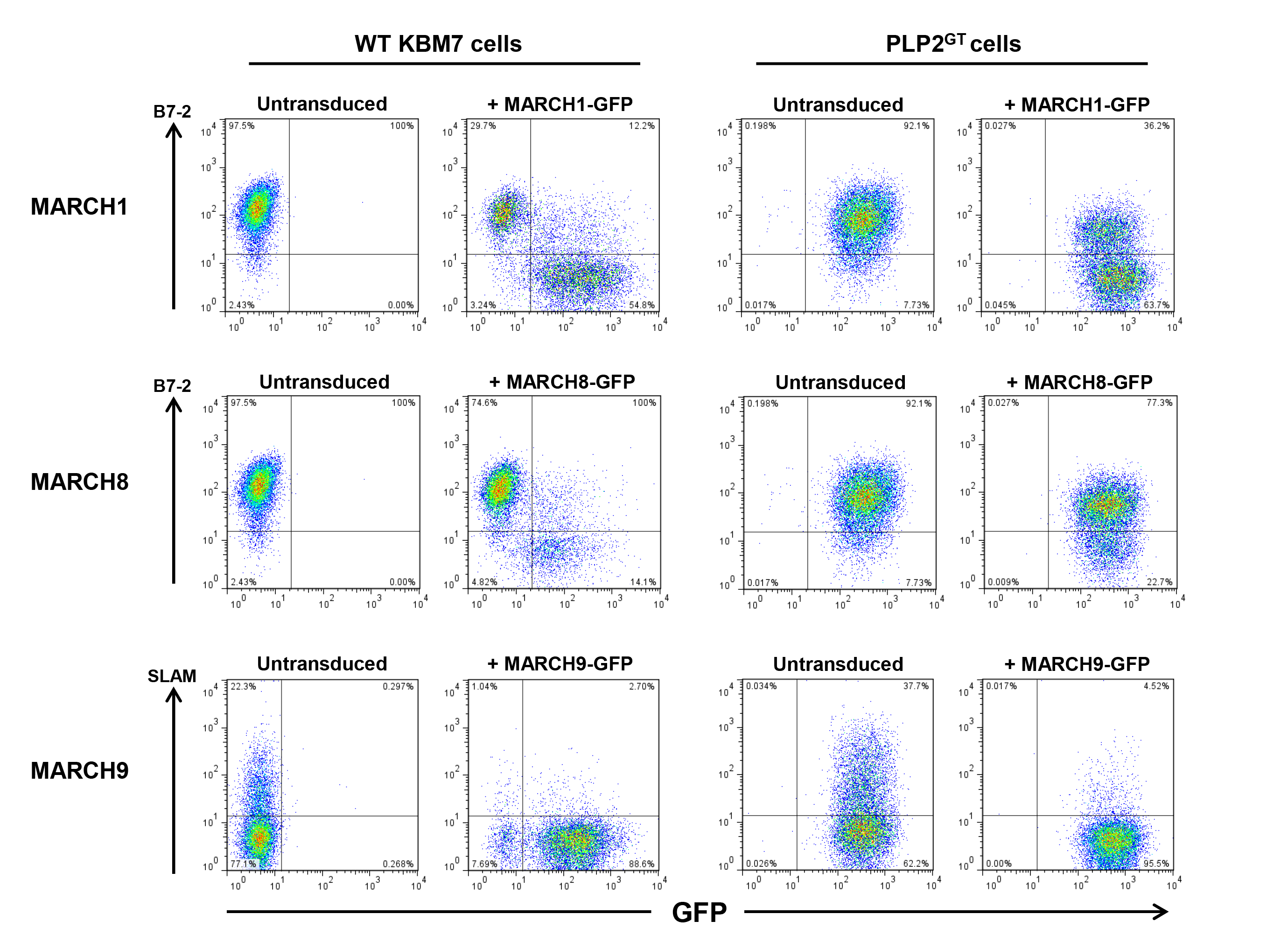

Supplement: Figure S4 — Downregulation of cell surface receptors by MARCH proteins is unaffected by loss of PLP2. Wild-type KBM7 cells or PLP2GT cells were transduced with lentiviral vectors encoding the indicated MARCH proteins along with Emerald-GFP, and the cell surface levels of the MARCH target proteins B7-2 (MARCH1 and MARCH8) and SLAM (MARCH9) were assessed by flow cytometry. (TIF) [file ppat.1003772.s004.tif]

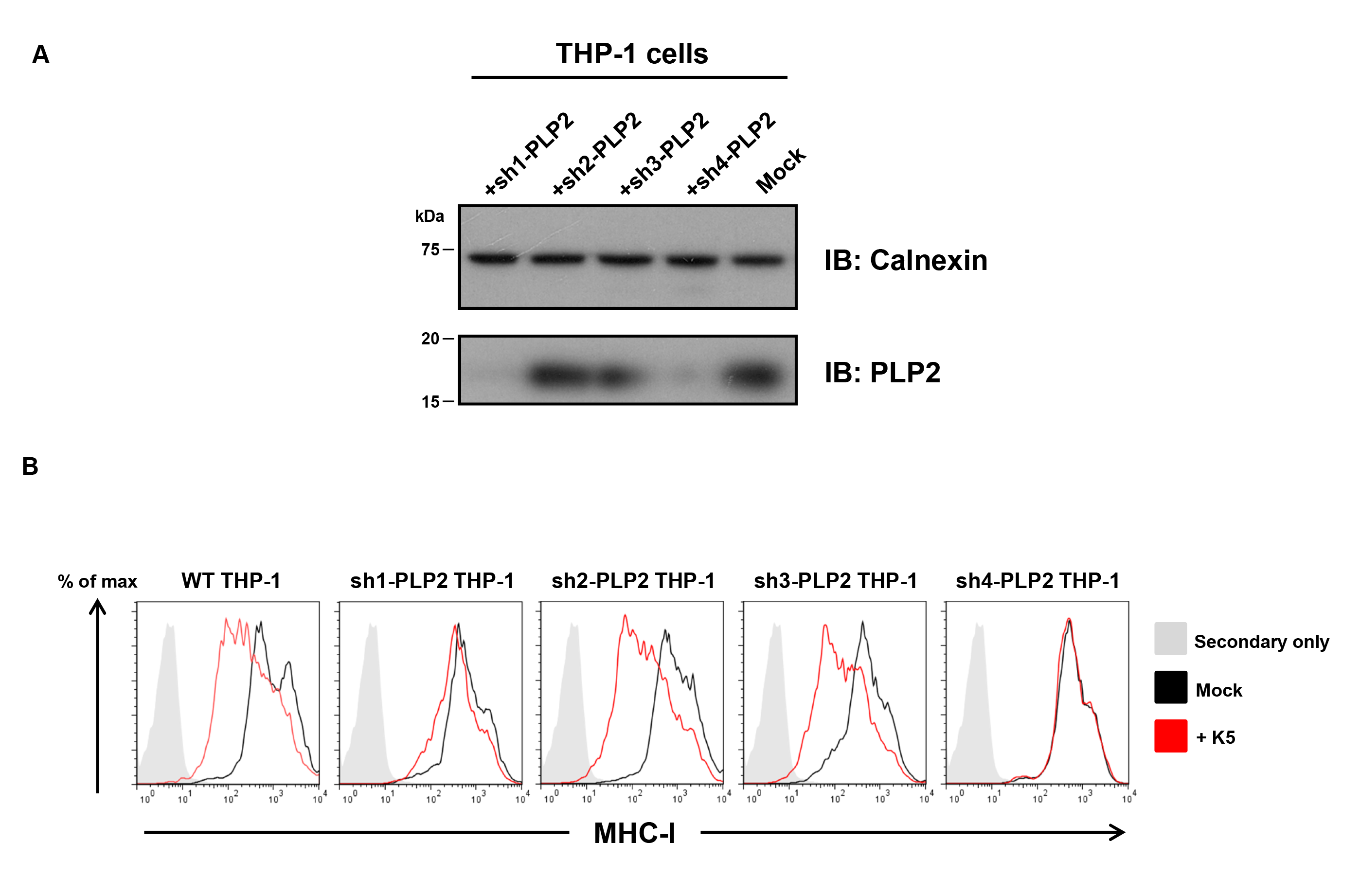

Supplement: Figure S5 — Validation of shRNA lentiviral vectors to knockdown PLP2 expression. (A) The effect of the shPLP2 vectors on PLP2 expression as assessed by immunoblot. THP-1 cells were transduced with four independent shPLP2 lentiviral vectors, untransduced cells removed by puromycin selection, and PLP2 expression examined by immunoblot. (B) The effect of the shPLP2 vectors on PLP2 expression as assessed by the inhibition of the K5-mediated downregulation of MHC-I. The shPLP2 THP-1 cells from (A) were transduced with a lentiviral vector expressing K5, and the K5-mediated downregulation of MHC-I assessed by flow cytometry. (TIF) [file ppat.1003772.s005.tif]

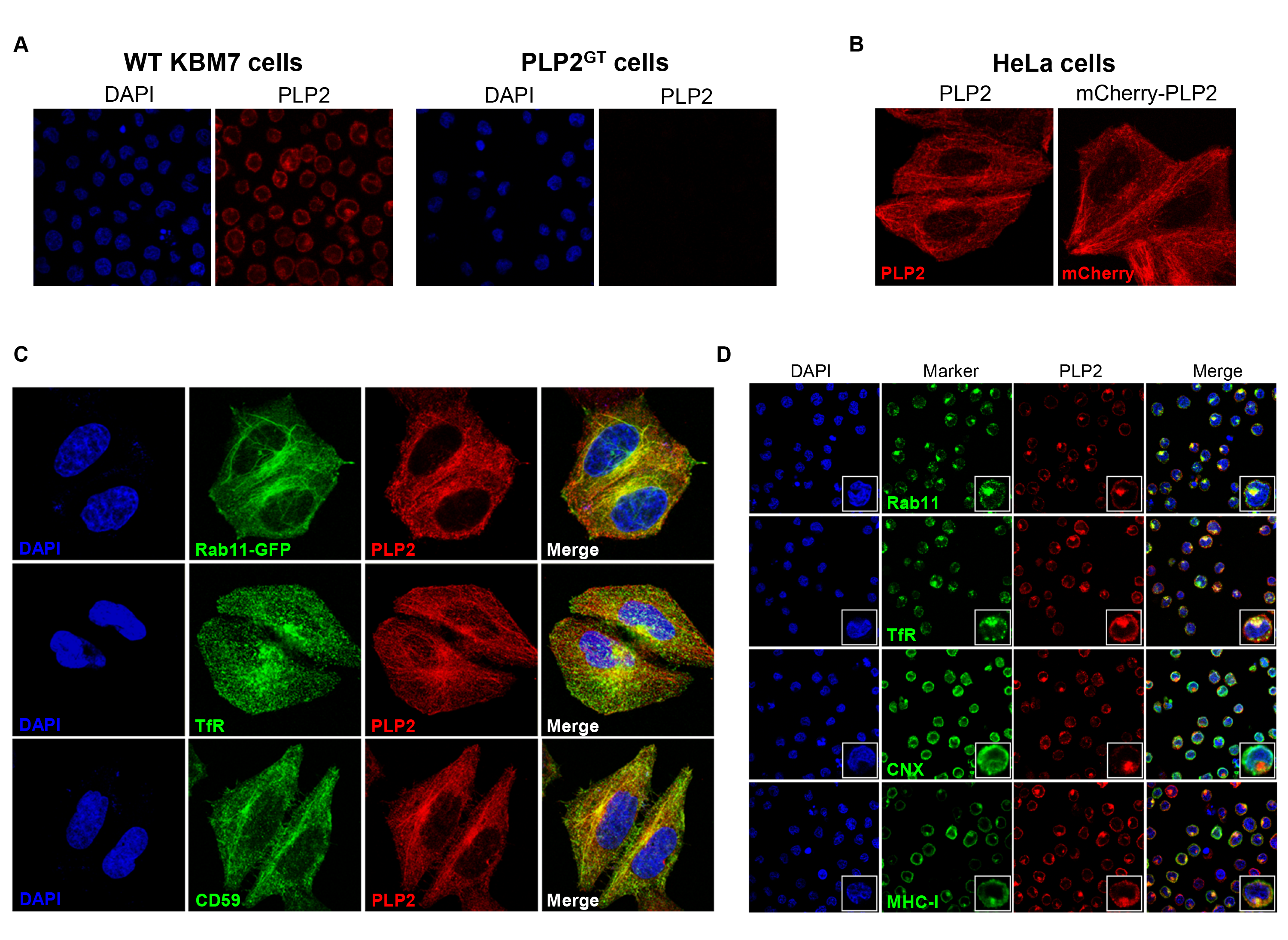

Supplement: Figure S6 — Subcellular localisation of PLP2. (A) Proving specificity of the PLP2 antibody for immunofluorescence. (B) Localisation of endogenous PLP2 and mCherry-PLP2 in HeLa cells. WT HeLa cells were either immunostained with the PLP2 antibody or transduced with a lentiviral vector encoding an mCherry-tagged PLP2. (C) PLP2 co-localises with recycling endosome markers in HeLa cells. HeLa cells were either transfected with a plasmid expressing Rab11-GFP or stained using mouse antibodies against the transferrin receptor (TfR) or CD59 (30 min antibody uptake) together with anti-mouse secondary antibodies conjugated to Alexa Fluor-488. PLP2 was stained using a rabbit antibody together with an anti-rabbit secondary antibody conjugated to Alexa Fluor-568. (D) PLP2 co-localises with recycling endosome markers in KBM7 cells. KBM7 cells were stained in a similar way for the indicated markers (CNX, calnexin). (TIF) [file ppat.1003772.s006.tif]

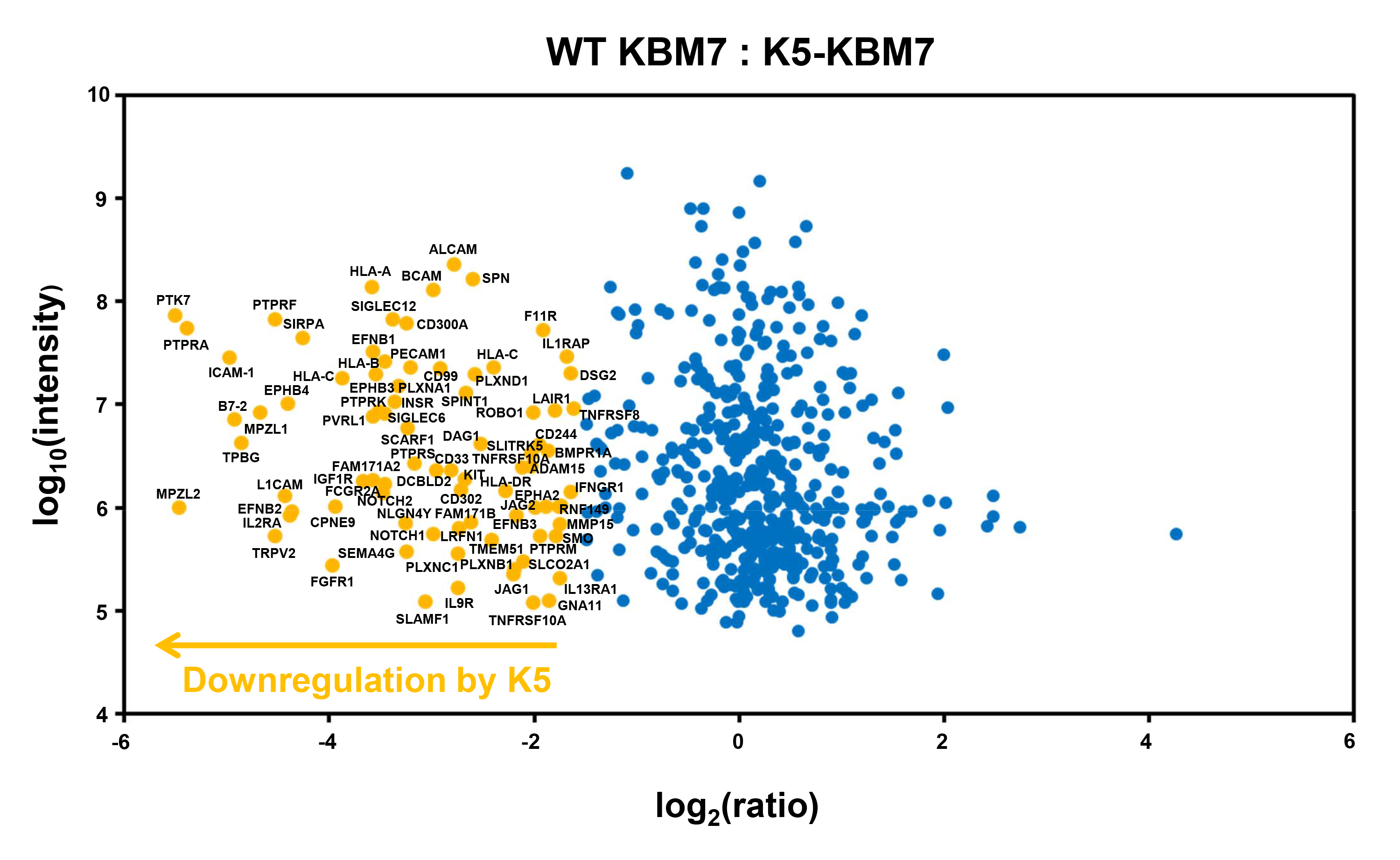

Supplement: Figure S7 — Plasma membrane profiling identifies many new K5 targets. A fully annotated version of Figure 7B is shown; proteins downregulated >3-fold from the plasma membrane in the presence of K5 are highlighted in orange. (TIF) [file ppat.1003772.s007.tif]

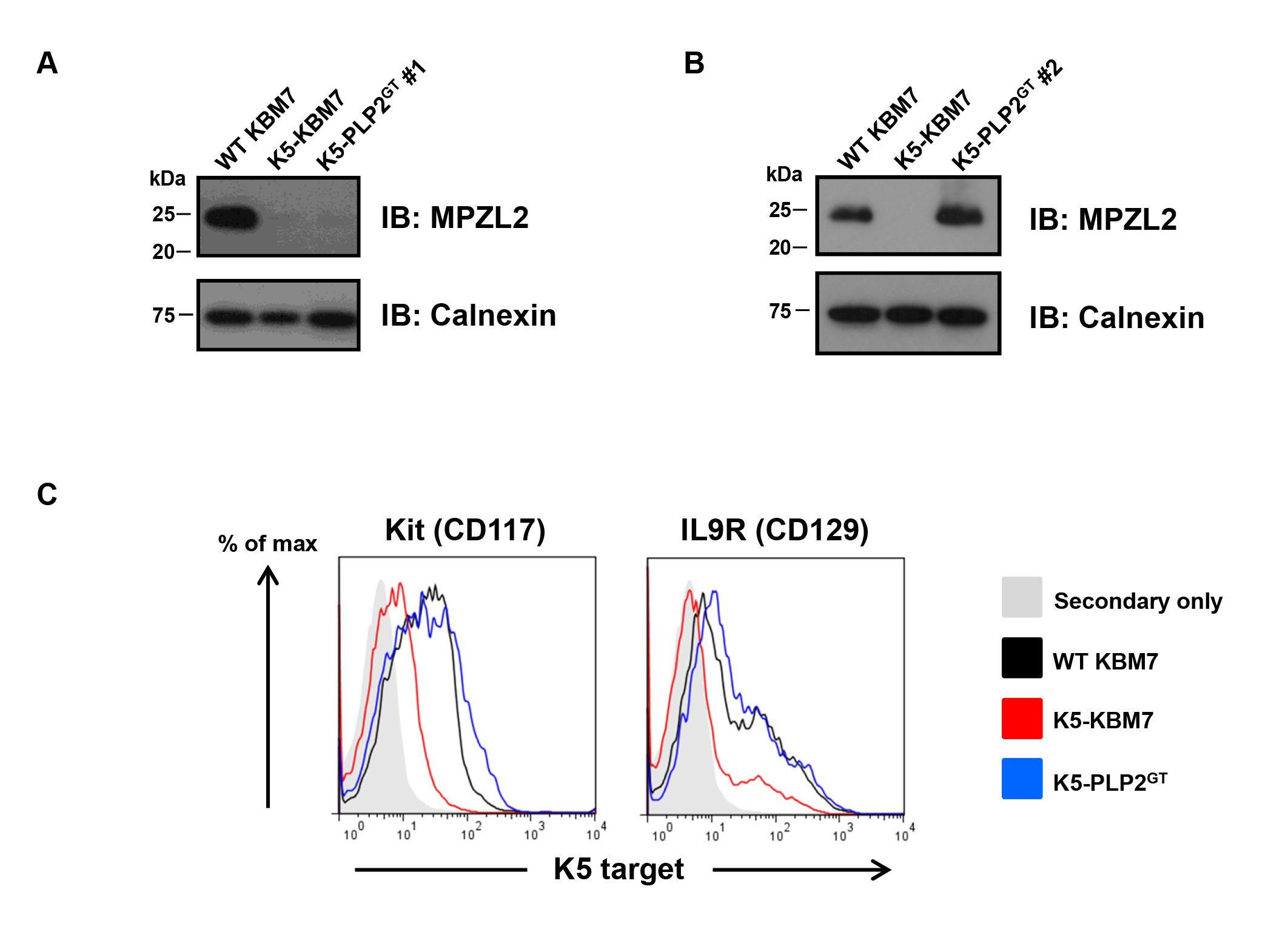

Supplement: Figure S8 — Examining potential PLP2-independent K5 targets. (A) Validation of the mass spectrometry data by immunoblot, confirming MPZL2 as a PLP2-independent K5 target in the K5-PLP2GT clone used. (B) MPZL2 cannot be degraded in the absence of PLP2 in an independent K5-PLP2GT clone. (C) Two additional putative PLP2-independent K5 targets identified, Kit and IL9R, were found to be in fact PLP2-dependent when examined by flow cytometry in an independent K5-PLP2GT clone. (TIF) [file ppat.1003772.s008.tif]
